# The Unsolved Problems of Milk Fever1Read at a meeting of the Veterinary Medical Association of New Jersey, held at Trenton, N J., January 9, 1902.

**Published:** 1902-04

**Authors:** J. M. W. Kitchen


### THE UNSOLVED PROBLEMS OF MILK FEVER.[1]

By J. M. W. Kitchen, M.D.

There are still some obstacles which stand in the way of the success of the up-to-date dairyman. It is determined that the high-power, special-purpose cow fed a narrowly balanced ration

[1] Read at a meeting of the Veterinary Medical Association of New Jersey, held at Trenton, N. J., January 9, 1902.

rich in protein is an essential to success in this day of keen competition and low prices for dairy products; but concomitant with the use of such a cow is the fact that the very best of them are very liable to be carried off by the disease which is popularly known as "milk fever," but which shall be designated more appropriately as parturient septicæmia.

As an introduction or text to the subject I will relate the history of the sickness of one of the best cows comprised in my Robinwood Farm herd of Jersey cattle, at Gilmanton, N. H.

Daisy of Gilmanton was a very healthy cow of about ten years of age, and weighing about 1000 pounds. The next prominent thing about her observed was that she was a big eater; her god was her belly. She was due to calve November 9th, and had been fed on a narrow feed well balanced with protein—as much in quantity as was necessary to keep her up to her flow. She was a 400-pound cow on ordinary good dairy feeding.

*October* 5, 1901. Her protein concentrates were stopped, and she was given a light feeding of poor English hay that was "broad" in its ratio of contained protein. She still picked up some pasturage. The idea was to dry her up for a few weeks before calving.

12*th*. She had given forty-seven and three-quarter pounds of milk during the past seven days, or nearly three and one-half quarts a day on an average.

13*th*. Took from her two and one-half quarts of milk—6 per cent. butter-fat; then the men apparently stopped milking her. I was absent from the farm at the time, else I do not think this cow would have died.

*November* 1*st*. Nine days before she was due to calve she began to make up her bag. It was larger than that of last year, 1900, but not any larger than in the years previous to 1900.

6*th*. Began to strip her, but got little milk.

9*th*. Due to calve, but no calf came to hand.

18*th*. Was apparently in good health; udder soft and pliable; got about two tablespoonfuls of milk from each teat; was a little constipated; gave her a little bran and a few doses consecutively of jalap powder. Her belly was very large, much more so than usual.

19*th*. Seemed ready to calve, but there were no labor pains. Began to give down milk; got three quarts at night. Calf was alive. Felt it move from outside.

20*th*. Seemed sick. No calf born. Gave her a large dose of salts at noon. Would not eat or drink. Seemed as if she had a bellyache, kicking at the belly. She dunged during the day.

Local veterinarian arrived at 10 P. M.; found the calf dead and os rigid, and concluded to leave the calf and see if the os would not relax. Found after-birth ripe. Said she was paralyzed with milk fever.

*21st.* Down flat on her belly, with her feet directly under her. Could not get her position changed. Did not dung during the past night. Slight labor pains, but not strong enough to expel the calf.

*November* 22, 1902. Mouth of the uterus much expanded. Took away the calf all right. The after-birth was expelled immediately afterward by the action of the uterus. No bad smell to it. Gave her a dose of salts, and injected in the uterus four quarts of carbolated water that had been boiled and cooled. Put her on the Schmidt treatment—*i. e.*, gave one injection of potassium iodide, three drachms, and water, one pint, boiled and cooled to 100° F. The injection was not repeated. Gave her another dose of salts. Had a rectal passage this night. Had no objectional vaginal discharge now or afterward.

*23d.* On her feet, and no signs of paralysis. Fed her on hay tea; would eat and drink a little, but acted as if her mouth was sore. Rectal discharges thin during the morning; got thicker and were scanty in the evening.

*24th.* Condition about the same. Little rectal discharge.

*25th.* Down flat. Seemed in great pain. Dosed her with hay tea and gave four ounces of whiskey every four hours. Began to have very offensive rectal discharges.

*26th.* Not so well, but still up. Seemed to be sinking away and losing strength. Would take no nourishment. Rectal discharges very offensive. Veterinarian diagnosticated inflammation of the bowels. Gave flaxseed tea.

*27th.* Down flat and would not get up. In great pain; seemed to have a convulsion.

*28th.* Seemed a little brighter. In the evening took a little water herself.

*29th.* About the same or a little better. Gave her a little oatmeal water.

*30th.* About the same. Would drink a little, but not eat. Continued to sink, and died at 1 A. M. in the night.

I will give you my interpretation of the case, and will ask your criticism and comment on the same.

The diagnosis, I think, should be that of a complication of delayed parturition, of a moderate attack of milk fever and congestion, and intestinal inflammation secondary to the absorption of ptomain poison.

The delay in calving, I think, was due to the fact that the cow was deprived of her accustomed protein nourishment, so that she did not have sufficient nerve force and uterine muscular strength to expel the calf. Possibly a gradual absorption of ptomains after the bag began to make up may have had some influence; possibly the fact of the cow having given milk so long had some influence.

The cow was not nourished actively enough during the attack. She should have had protein nourishment. The whiskey alone was adding a poison to a poison. The hay tea had no protein in it. The milk fever was due, I think, to the fact that the old milk was left in the udder from October 13th to November 6th, and became a breeding medium for bacteria. The watery elements were absorbed, but left a ferment there that could not be stripped out. Possibly the parenchyma of the udder became infiltrated with the germs, and when a new supply of blood was directed to the udder at the time when the placenta became ripe, and practically dissociated from the uterine walls, the ferment germs were nourished into proliferating activity, and the ptomain poison was absorbed into the system.

We infer that milk fever, so-called, is due to the action of a ptomain poison, because the symptoms are so like those in human patients. We believe that in most cases the route of infection is through the udder, because the trouble hardly ever occurs with the first calf and seldom with the second calf. It is only in mature large producers where there are large milk ducts that the disease is likely to occur. In such cows germs can quite readily find entrance through the large milk ducts of the teats.

We also think the udder is the route of infection because in most cases the Schmidt treatment of injecting and rubbing in the iodide of potassium solution, if thoroughly well done, saves the cow. It is, of course, a possible thing for the infection to be through the uterus; but this is probably rare, as the young animal would be as likely as the old to be thus infected. Furthermore, it seems to be warded off when a quick, full flow of blood to the udder is prevented in various ways, or at least is only slight in its effect.

I think it is about time that the world of science gave us definite information in regard to this problem of infection. Is it due to the old milk in the udder? Is it due to the slow accumulation of the new formation of albumins and fatty colostrum becoming a medium for the growth of ferment germs? Do the germs involve the parenchyma of the udder, and, if so, is it a fatal condition? We can imagine that if the germs gain access to the blood the result will be immediately fatal.

What we want is that the dairy bacteriologists should make laboratory examinations of cows that have the fever. We want the stations to experiment in washing out the udders of cows every week during the time between drying up and calving. Will not those here present use their influence to get such work done?

The defects in the treatment of this cow were in not giving her a reasonable amount of protein feed up to within ten days to a week before calving. When milk fever was reported her udder should have been immediately washed out with either salt solution or with a solution of sodium sulphite or other antiseptic. The Schmidt treatment should have immediately followed, and it should have been repeated every two hours while in danger, and she should have been actively supported with drenches of milk gruel.

---

COLD IN THE ETIOGENESIS OF PNEUMONIA. Bourgés, veterinarian in the French army, made observations in the winter of 1900–1901, during the Chinese expedition. The months of December, January, and February in Northern China are very cold, and it seldom snows or rains. The atmosphere is dry. The expedition carried 2850 horses and mules, unacclimated, and collected from Europe, Algeria, Australia, Corea, and China. Most of them were kept in poorly constructed stables, with a temperature of from 10° to 15° below zero; others were sheltered at a temperature of from 2° to 3° below zero; the ventilation was excessive. The mortality from intrathoracic diseases during these months was 7. These diseases were rare, because the horses were well nourished, moderately worked, and not exposed to the unhealthful influences of crowded stables and an impure atmosphere. On the other hand, during the months of November, March, and April, when the temperature was more moderate, the mortality was 10.

The conclusions were drawn: That intrathoracic diseases are no more frequent in winter than during other seasons; that a cold and dry atmosphere is not a causative factor in pneumonia; that these facts militate against the crowding of horses in poorly ventilated and unsanitary stables.—*Rec. de Méd. Vét.*

